# Anorexia Nervosa and Emotional Dysregulation: A Longitudinal Study on the Characteristics and Clinical Implications in a Group of Female Adolescents

**DOI:** 10.3390/children13030402

**Published:** 2026-03-14

**Authors:** Pamela Fantozzi, Chiara Covelli, Francesca Ditaranto, Fabio Apicella, Vittorio Belmonti, Raffaella Tancredi, Valentina Levantini, Sara Calderoni

**Affiliations:** 1 IRCCS Fondazione Stella Maris, Viale del Tirreno 331, I-56018 Pisa, Italy; chiara.covelli@fsm.unipi.it (C.C.); francesca.ditaranto@fsm.unipi.it (F.D.); fabio.apicella@fsm.unipi.it (F.A.); vittorio.belmonti@fsm.unipi.it (V.B.); raffaella.tancredi@fsm.unipi.it (R.T.); valentina.levantini@fsm.unipi.it (V.L.); sara.calderoni@fsm.unipi.it (S.C.); 2 Department of Clinical and Experimental Medicine, University of Pisa, 56126 Pisa, Italy

**Keywords:** anorexia nervosa, emotional dysregulation, female adolescents

## Abstract

**Background**: Anorexia nervosa (AN) is a severe eating disorder occurring most frequently in adolescence, characterized by a high prevalence of psychiatric comorbidity. Emotional dysregulation (ED) refers to a transdiagnostic construct that often drives disordered eating behavior. The present study aimed to evaluate differences and similarities in the clinical presentation and response to treatment of young AN patients with high levels of ED (AN+ED) and with low levels of ED (AN−ED). **Methods**: A total of 40 female inpatients aged between 12 and 18 years were consecutively recruited and subdivided into two groups (AN+ED: *n* = 21; AN−ED: *n* = 19), based on the median of the subscale Affective Instability (AI) of the Reactivity, Intensity, Polarity and Stability questionnaire—youth version (RIPoSt-Y). At the recruitment (T0), and after 6 months (T1), the Body Mass Index (BMI) was calculated, and questionnaires and scales were administered to assess (a) the general functioning; (b) the severity of the eating disorder; and (c) the associated psychopathology. **Results**: At T0, an independent-samples *t*-test showed that the AN+ED group was characterized by a significantly greater impairment in clinical functioning and a greater severity of both the eating disorder and the associated psychopathology compared to the AN−ED group. At T1, the AN+ED group also showed significantly higher levels of cyclothymic, depressive, and anxious symptoms than the AN−ED group. Moreover, repeated-measures ANOVAs revealed a statistically marked improvement over time of the bulimic behaviors in the AN+ED group only. **Conclusions**: The present study underscored distinctive clinical features in AN patients with high and low levels of ED. Specifically, the AN+ED group was characterized by a most likely severe clinical phenotype that requires tailored intervention strategies.

## 1. Introduction

Anorexia nervosa (AN) is a severe and frequently chronic eating disorder with a peak incidence in adolescence [1,2]. AN is associated with a significant resistance to treatment and the highest mortality rate among psychiatric disorders [3,4]. According to the Diagnostic and Statistical Manual of Mental Disorders, 5th Edition, Text Revision [5], AN is defined by restriction of energy intake, leading to a significantly low body weight; intense fear of gaining weight or becoming fat, or persistent behaviors that interfere with weight gain; and disturbance in the way one’s body weight or shape is experienced or persistent lack of recognition of the seriousness of the low body weight. The DSM-5-TR distinguishes between the restrictive type (AN-R), where weight loss is achieved through caloric restriction, fasting, and/or excessive physical activity, and the binge-eating/purging type (AN-B/P), where restrictive behaviors are accompanied by binge eating or purging behaviors (e.g., self-induced vomiting) [5].

A great amount of scientific studies document high rates of psychiatric comorbidity in AN, especially mood disorders, major depressive disorders, and anxiety disorders [6,7,8], also during adolescence [1,9]. Recent reviews emphasize the presence of global difficulties with regulating affective states in subjects with AN [10,11], and more broadly in adolescents with the spectrum of eating psychopathology [12]. The model proposed by Haynos and Fruzzetti [13], also supported by other authors [14], suggests that patients with AN could use low-calorie diet, binge eating, purging, or physical activity to regulate aversive emotional states in conflict situations. Some evidence suggests that subjects with AN show heightened sensory sensitivity [15], problems in emotional clarity and emotional awareness [16], a lower ability to regulate emotions when they are upset [17], problems in regulating positive affective states [18], difficulties in accepting emotional experiences [19], impaired empathy skills [20,21], and interoceptive deficits [22].

The term emotion regulation refers to any strategy that modulates emotion unfolding and shapes the resulting reactions, both emotionally and behaviorally [23,24,25]. Emotional dysregulation (ED), to date widely recognized as a transdiagnostic dimension, is characterized by mood lability, emotional reactivity, impulsivity, and overt irritability [26]. The multidimensional model by Gratz and Roemer [27] identifies the main characteristics of ED: problems in the understanding and in the acceptance of emotions, difficulties in controlling impulsiveness, and a deficit of functional strategies to modulate emotional responses in order to fulfill personal goals and situational demands. In a review on ED in eating disorders, Lavender et al. [11] identified broad emotion regulation problems, with a deficit in emotion regulation across the four dimensions in AN individuals. Moreover, ED has been consistently linked to the development and maintenance of eating disorders [28,29]. In this context, a recent UK longitudinal population-based cohort study, involving a total of 15,896 participants, detected that difficulties in developing emotion regulation skills in childhood were associated with an increased risk for anorexia nervosa and atypical anorexia nervosa in adolescence [30]. Most previous studies regarding ED in AN were conducted on adult samples [31,32] and analyzed the response to psychological treatments (for review, see [10]).

Based on this background, the aims of the current study were (1) to investigate similarities and differences in symptom profiles of young female inpatients with AN regarding the levels of ED, and (2) to evaluate symptom variation after six months from recruitment. Despite the growing interest in the scientific literature for ED in eating disorders, to our knowledge, this is the first study examining this construct in a group of adolescent females with AN.

## 2. Materials and Methods

### 2.1. Participants

Forty female adolescent inpatients with AN—comprising the restricting type (AN-R, thirty-six) and binge-eating type (AN-B/P, four)—were recruited at the Eating Disorders Unit of the IRCCS Fondazione Stella Maris (Pisa, Italy), a tertiary care university hospital. Patients were selected from all consecutive participants admitted to the unit from June 2024 to June 2025. Eligibility criteria were defined as follows: diagnosis of AN-R or AN-B/P, according to the Diagnostic and Statistical Manual of Mental Disorders, 5th Edition, Text Revision (DSM-5-TR criteria) [5]; female sex; age from 12 to 18 years; Full-Scale Intelligence Quotient equal to or greater than 85. The exclusion criteria were the same we used in another previous work [33]: psychotic symptoms; neurological disorders or sensory deficits; current or history of substance abuse; medical conditions not correlated with the eating disorder; and significant intrinsic instability requiring constant medical care supervision. Diagnostic procedures for AN were carried out by a multidisciplinary team that included a child/adolescent neuropsychiatrist. The mean age was 14.43 years (range: 12–18, SD = 1.50). The mean duration of illness was 12.64 months (range: 11–17 months, SD: 5.83). The mean Body Mass Index (BMI) at admission (T0) was 16.11 kg/m^2^ (range: 12.55–21, SD: 2.08). Thirty-nine patients (97.50%) fulfilled the criteria for an Axis I mood and/or anxiety disorder. Thirty-four patients (85.00%) received psychopharmacological treatment with selective serotonin reuptake inhibitors (Fluoxetine, Sertraline, Fluvoxamine, Paroxetine) and/or atypical antipsychotics (Risperidone, Aripiprazole, Olanzapine, Quetiapine) and/or mood stabilizers (Lithium Salt, Gabapentin); nineteen patients (47.50%) received a combined psychopharmacological therapy. The remaining six subjects were medication-naïve. All patients underwent individual psychotherapy, and twenty-three patients (57.50%) underwent family psychotherapy too. Further details regarding the pharmacological treatments and the psychotherapy interventions are shown in Table 1.

Informed consent was obtained from all subjects involved in the study and their parents. The study was conducted in accordance with the Declaration of Helsinki and approved by the Institutional Review Board of the Meyer Hospital (Florence, Italy; Regional Pediatric Ethics Committee of Tuscany, code TerapiaAN24, approval date: 19 June 2024).

### 2.2. Measures

Patients underwent a full diagnostic assessment, including self-rated clinical questionnaires and the semi-structured interview Kiddie Schedule for Affective Disorders and Schizophrenia, present and lifetime version [34], administered by trained child psychiatrists, as well as a neuropsychological evaluation based on Wechsler Intelligence Scale for Children—fourth edition [35] or on the Wechsler Adult Intelligence Scale—fourth edition [36]. Clinicians also recorded Body Mass Index (BMI) and relevant clinical information, including the type of pharmacological interventions.

The following clinician and patient-rated scales and questionnaires, some of which previously used in other studies by our group [37], were administered:

- The Clinic Global Impression—Severity (CGI-S) [38]: A global scale completed by the clinician that assesses the overall severity of the disorder, regardless of its psychopathological complexity;
- The Children’s Global Assessment Scale (C-GAS) [39]: A clinician-completed scale that evaluates the level of functional impairment;
- The Reactivity, Intensity, Polarity, and Stability—youth version (RIPoSt-Y) [40,41]: A 31-item questionnaire aimed to assess the ED along three main dimensions (Affective Instability—AI, Emotional Reactivity—ER, and Interpersonal Sensitivity—IS) with corresponding cutoff scores and good validity and reliability;
- The Cyclothymic-Hypersensitive Temperament (CHT) [42,43]: A 22-item self-assessment questionnaire employed to identify cyclothymic temperamental traits in young people aged 10 or older;
- The Youth Self Report—for youths aged 11 to 18 years (YSR-11/18) [44]: A widely used, reliable, and validated child-report measure that assesses problem behaviors along three broadband scales (Internalizing—Int, Externalizing—Ext, and Total Problems) and several empirically based syndromes and DSM-oriented scales; we evaluated the score of the Anxious/Depressed scale (A/D) and of the Withdrawn/Depressed scale (W/D);
- The Eating Disorder Inventory—third edition (EDI-3) [45,46]: A 91-item self-assessment questionnaire routinely employed in the diagnosis of eating disorders with good validity and reliability; we examined the three eating disorder subscales (Drive for Thinness—DT, Bulimia—B, and Body Dissatisfaction—BD) and two of the nine psychological trait subscales (Interoceptive Deficits—ID and Emotional Dysregulation—ED);
- The Body Uneasiness Test (BUT) [47,48]: A 71-item self-report questionnaire designed to assess body image with good psychometric properties; we evaluated the subscales Weight Phobia—WP and Body Image Concerns—BIC.

### 2.3. Statistics

Statistical analyses were performed with IBM SPSS Statistics for Windows, Version 26.0. Based on the median of the subscale AI of the RIPoST-Y, the sample was divided into two groups: anorexia nervosa with high levels of emotional dysregulation (AN+ED) and anorexia nervosa with low levels of emotional dysregulation (AN−ED).

This approach was adopted to identify subgroups with relatively higher versus lower levels of emotional dysregulation within the clinical sample. Notably, the median AI score (57) in the present sample was slightly above the proposed cut-off (55), indicating generally elevated levels of emotional dysregulation across participants.

Independent-samples *t*-tests were conducted to test differences at T0 between the AN+ED and AN−ED groups for anthropometric measurements, cognitive profile, and questionnaire scores. A chi-square (χ^2^) test was used to examine baseline differences between the two groups according to ongoing pharmacological treatment (antidepressant, antidepressant +/− atypical antipsychotics, mood stabilizers +/− antidepressant +/− atypical antipsychotics, no psychopharmacological therapy).

Repeated measures ANOVA was conducted to assess the general functioning (CGI-S, C-GAS), the severity of the eating disorder (EDI-3 DT, EDI-3 B, EDI-3 BD; BUT-WP, BUT-BIC), and the associated psychopathology (EDI-3 ID, EDI-3 ED; CHT; YSR 11/18 Int, YSR 11/18 Ext, YSR 11/18 A/D, YSR 11/18 W/D) at the start of medication (T0) and at 6-month follow-up (T1). The effect of time, group, and time x group interaction was tested.

Unless otherwise specified, the level of significance was set at *p* < 0.05 (two-tailed).

## 3. Results

We divided the participants into two groups (AN+ED: *n* = 21; AN−ED: *n* = 19), based on the median of the subscale AI of RIPoSt-Y. Independent *t*-test analysis indicated a significant difference between the AN+ED and the AN−ED groups in age (t = 2.195; *p* = 0.034). The AN+ED group showed significantly lower scores on the Working Memory Index of the intelligence tests than the AN−ED group (*p* = 0.035). No significant differences were detected for the other indexes or for the Full-Scale Intelligence Quotient. The AN+ED group was characterized by (a) a significantly greater impairment of the clinical functioning (CGI-S: *p* = 0.003; C-GAS: *p* = 0.013) with a large effect size; (b) a significantly greater severity of the eating disorder (BMI: *p* = 0.038; EDI-DT: *p* = 0.028; EDI-B: *p* = 0.013; EDI-BD: *p* = 0.010; BUT-WP: *p* = 0.013; BUT-BIC: *p* = 0.010) with a large effect size for BUT-WP and BUT-BIC; (c) a significantly greater severity of the associated psychopathology (EDI-ID: *p* < 0.001; EDI-ED: *p* < 0.001; CHT: *p* < 0.001; YSR-Int: *p* = 0.005; YSR-Ext: *p* = 0.001; YSR-A/D: *p* = 0.002; YSR-W/D: *p* = 0.025) with a large effect size for all the subscales, except for the YSR-W/D subscale compared to the AN−ED group. Moreover, at T1, the AN+ED group showed significantly higher scores on the EDI-ID (*p* = 0.003) and on the EDI-ED (*p* < 0.001) compared to the AN−ED group, with a large effect size. Similarly, the AN+ED group showed significantly higher scores on CHT (*p* < 0.001), YSR-Int (*p* = 0.022), YSR-Ext (*p* < 0.001), and YSR-A/D (*p* = 0.008) compared to the AN−ED group, with a large effect size for all the subscales, except for the YSR-Int subscale. Further details are shown in Table 2.

No significant differences in pharmacological interventions were observed between the two groups, as assessed by the chi-square (χ^2^) test (χ^2^ (3) = 3.805, *p* = 0.283).

Repeated-measures ANOVAs, with age included as a covariate, revealed a significant effect of time on BMI (*p* = 0.037), CGI-S (*p* = 0.038), C-GAS (*p* = 0.008), and BUT-BIC (*p* = 0.023), as well as a significant group effect on C-GAS (*p* = 0.030), EDI-B (*p* = 0.025), EDI-ID (*p* < 0.001), EDI-ED (*p* < 0.001), BUT-WP (*p* = 0.018), and BUT-BIC (*p* = 0.032). Of particular relevance, we observed a significant time × group interaction on EDI-B (*p* = 0.046), indicating a marked improvement over time of the score of the EDI-B subscale only in the AN+ED group. Further details are shown in Table 3.

## 4. Discussion

This is an exploratory and preliminary study, which aimed to investigate shared and distinctive features in terms of clinical, neuropsychological, and psychopathological features among AN patients with high and low levels of emotional dysregulation, consecutively recruited in a tertiary care university hospital. We also evaluated course variation after six months of multidisciplinary treatment.

We divided the subjects into two groups (AN+ED and AN−ED) based on the median of the Affective Instability (AI) subscale of the RIPoSt-Y [41]. Compared to the other two scales of the RIPoSt, the AI subscale evaluates the presence of a cyclic pattern of rapid mood swings of opposite polarities, which may compromise the functioning of the subject both from a social and performance perspective [41]. We selected this subscale considering the high prevalence of affective dysfunction [49,50], including bipolar spectrum disorders [51,52,53], in individuals with eating disorders.

The AN+ED group was significantly younger than the AN−ED group. Recent studies support the hypothesis that eating behaviors among children and adolescents would be influenced by ED [54,55]. In turn, ED is affected by early relational patterns, including emotional neglect, overcontrol, and inconsistent parental responses, which may represent a possible target for primary prevention in eating disorders [56,57,58]. Especially among young children, research suggests that typical responses to stress or mood states may consist of decreased appetite [59] or excessive eating [60]. These data are in line with the difference in age we detected in our sample.

In our study, there is a stronger prevalence of subjects with AN restricting type than the binge/purging type. Previous investigations reported that patients with AN-B/P had not only emotion regulation deficits compared to patients with AN-R [61,62], but also less adaptive and more maladaptive strategies to regulate emotions [63]. On the other side, some Authors suggested that AN-R patients would present more deficits in emotion expression and inhibition and emotional overcontrol than AN-B/P patients [31]. However, findings indicating that the two subtypes of AN did not significantly differ in emotion regulation profile from each other also emerged [61].

The AN+ED group showed significantly lower scores on the Working Memory Index (WMI) of the intelligence tests than the AN−ED group. The WMI assesses the capacity to hold and manipulate information in mind, both verbal and visual. Prefrontal cortex executive functions, including WM, interact with limbic processes to foster impulse control. Brooks et al. [64] examined the role of WM for cognitive control. The Authors hypothesized that higher WM ability may support the neural process of excessive epistemic foraging, likely cognitive sampling of the environment to test predictions about the world, which reduces distraction from salient stimuli. On the other hand, lower WM capacity may be associated with higher impulsivity and reduced epistemic foraging. Based on this model, the AN+ED group may present reduced skills of attention, short-term memory, and processing speed than the AN−ED group, as well as more impulsive behaviors.

At T0, the AN+ED group was characterized by a significantly greater impairment of the global functioning, as measured by the clinician-completed CGI-S and C-GAS scales. We did not find a significant difference in the CGI-S and in the C-GAS between the two groups at T1. Clinical severity assessment based on the CGI-S scale reveals marked severity of symptoms with high interference with daily life functioning. Similarly, mean scores in C-GAS identify the presence of meaningful deficits in global functioning in almost all areas of daily life, such as social functioning.

At T0, the AN+ED group showed a greater severity of the eating disorder, with a significantly lower BMI, than the AN−ED group. This data highlights the importance of closely monitoring weight in patients with AN and high levels of ED. We found significantly lower scores on the three EDI-3 eating subscales (i.e., Drive for Thinness, Bulimia, and Body Dissatisfaction), which assess the attitudes toward weight, body shape, and eating [45,46]. We also detected higher scores on the Weight Phobia and Body Image Concerns subscales of the BUT, evaluating the fear of becoming fat and the worries related to physical appearance, respectively [47,48]. We did not find at T1 significant differences in the indices evaluating the severity of the eating disorder between the two groups. Our results are in line with recent research suggesting that the development and maintenance of AN are supported by emotion regulation deficits [19,65,66]. In addition, a meta-analysis on this topic documented that a lack of adaptive emotion regulation strategies—particularly difficulties in problem-solving skills, and greater use of avoidance, rumination, and suppression—were linked to worse eating disorder psychopathology [67]. In our study, the AN+ED group presented high levels of body uneasiness as measured with the two subscales of the BUT. Studies indicate that body dissatisfaction begins to increase as children get older and poor body esteem increasingly becomes a risk factor for a variety of unhealthy behaviors, including dieting and disordered eating during adolescence [68]. In a recent study, Shriver et al. [55] highlighted the association between emotion regulation and negative body image in children and dysregulated eating in late adolescence.

At T0 and T1, we found a significantly greater severity of the associated psychopathology in the AN+ED group than in the AN−ED group, as measured by the Interoceptive Deficits and Emotional Dysregulation subscales of the EDI-3. We decided to examine these two subscales because they evaluate the difficulty associated with recognizing and responding to emotional states and the tendency to emotional instability, impulsivity, recklessness, anger, and self-destructiveness [45,46]. Among psychiatric comorbidities, at T0, cyclothymic disorder, externalizing and internalizing symptoms, and depressive and anxiety symptoms (measured by the CHT, the Externalizing and the Internalizing scale of the YSR, and the Withdrawn/Depressed and the Anxious/Depressed subscales of the YSR), were significantly more prevalent in the AN+ED group than in the AN−ED group. At T1, we found the same significant differences between the two groups, except for the Withdrawn/Depressed subscale of the YSR. The results of this research are congruent with the studies suggesting a link between emotion regulation and eating disorder psychopathology [13,69]. Wildes and colleagues [70] found that the relationship between depressive symptoms and eating disorder psychopathology in AN was partially mediated by emotion avoidance, suggesting that the regulation of negative mood states may be partially due to eating disorder symptoms. Our results are also in line with a recent study in which female adolescents with non-suicidal self-injury (NSSI) and feeding or eating disorders (FEDs) displayed a greater prevalence of cyclothymic disorder and a more severe clinical presentation compared to peers with NSSI or FED only [37]. Particularly, cyclothymic disorder is known to be characterized by emotional dysregulation [71], affective reactivity, and interpersonal sensitivity [72]; these aspects can lead patients to a longer duration and intensity of their emotional experiences, which are consequently perceived as a continuous source of suffering. Moreover, a study confirmed that FED behaviors have a function in regulating emotions, including feelings of shame, especially body shame [73].

Repeated-measures ANOVAs, with age included as a covariate, revealed a significant effect of time on BMI. In acute AN, the relationship between low body weight and emotion regulation difficulties remains unclear. In a sample of patients with acute AN, Brockmeyer and colleagues [74] found that lower Body Mass Index was associated with fewer emotion regulation difficulties. Racine and Wildes [19] suggested that depressive symptoms influenced changes in emotional regulation difficulties and AN symptomatology. Haynos and colleagues [61] found that difficulties with emotion regulation did not improve with weight restoration through specialized inpatient treatment, although significant improvements in both general psychological distress and specific eating disorder psychopathology were found. Conversely, Rowsell et al. [31] observed an improvement in emotion regulation difficulties in the subgroup of AN subjects who became weight-restored.

From a statistical point of view, we detected a marked improvement over time of the bulimic conduct as measured with the Bulimia subscale of the EDI-3, only in the AN+ED group. Considering the link between difficulties in emotion regulation and impulsivity, we could speculate that the reduction in functional interference related to emotional difficulties—at least partly attributable to a combination of pharmacological and psychotherapeutic treatments—could improve impulsive behaviors and consequently the bulimic symptoms in AN subjects with high levels of ED.

The two groups did not show significant differences in the pharmacological treatments as measured by the chi-square (χ^2^) test. The current literature on the pharmacological treatment of patients with FEDs is still controversial, also in light of the possible adverse effects linked to the clinical impairment of these patients. Antidepressants, particularly the selective serotonin reuptake inhibitors, received the greatest attention and may represent the treatment of choice for AN and AN-related depressive or anxiety symptoms [75]. Second-generation antipsychotics are frequently prescribed for the treatment of resistant anorexia nervosa. In a recent review, Thorey et al. [76] documented that Olanzapine was the treatment most frequently prescribed and that other atypical antipsychotics, such as aripiprazole, quetiapine, and risperidone, were well tolerated with mild and transient adverse effects in this population. Regarding the use of mood stabilizers in anorexia nervosa, Lithium and other mood stabilizers showed mixed evidence of efficacy, with the former promoting significant improvements in weight gain but with some degree of uncertainty [77]. As far as non-pharmacological treatments, it is important to highlight that, given the clinical severity of the patients involved in the study, all of them underwent individual psychotherapy, and most of them underwent family psychotherapy too.

Our findings add to growing evidence suggesting that anorexia nervosa with emotion regulation difficulties represents a specific phenotype, characterized by an overall important clinical severity. Previous research and the findings of the current study suggest that the critical window for optimizing emotion regulation skills, promoting positive body image, and minimizing body dissatisfaction appears to be during childhood and early adolescence [78]. Considering the key role of emotion regulation deficits in both the development and maintenance of AN, integrating strategies to address these aspects may be important to improving treatment outcomes in AN.

The current study had a number of limitations. Firstly, the relatively small number of patients per group, along with the elevated number of statistical tests, could have reduced the statistical power of the analyses and increased the risk of type I and type II errors. Therefore, the findings and any derived clinical implications should be interpreted with caution. Future studies with larger samples could confirm our approach and allow for more robust conclusions. Secondly, participants were consecutively recruited from a single tertiary care center, and are homogeneous in terms of primary diagnosis and sex. Furthermore, our study did not include a healthy control group. These aspects are undoubtedly a strength of the study; however, they also limit the generalizability of the findings to broader clinical settings, including outpatient populations and male patients. Thirdly, the significant difference in age between the two groups would represent a potential confounding factor; therefore, age was included as a covariate in the longitudinal analyses. Fourthly, the use of a median split to define ED groups may have reduced the statistical power and limited our ability to capture the full variability of emotional dysregulation in our sample. Fifthly, the use of self-report questionnaire measures to assess the eating disorder behaviors and the associated psychopathology could result in reporting bias, particularly in a population characterized by limited insight and emotional awareness [79]. Nonetheless, the self-reported and parent-reported questionnaires employed in this study, together with the clinical assessment and semi-structured interviews, allowed us to permit a multi-informant and multidimensional investigation of the patients. Finally, the heterogeneity of the drug therapies used and the absence of detailed control for treatment intensity or psychotherapeutic interventions are also limitations. Therefore, future studies are needed to validate the current data.

## 5. Conclusions

In summary, this study supports the hypothesis that patients diagnosed with AN and high levels of ED exhibit distinct clinical features and responses to treatments as compared to patients with AN and low levels of ED. In our sample, the AN+ED group showed lower working memory abilities, marked global impairment of functioning, and significantly greater severity of the eating disorder and of the associated psychopathology than the AN−ED group. In AN subjects with high levels of ED, at the reevaluation after six months of multidisciplinary treatment, we also found a statistically significant improvement in the score of the EDI—Bulimia subscale, which evaluates thoughts and behaviors related to binge eating. We could speculate that, considering the ED as a transnosographic dimension across developmental psychopathology, the presence of high levels of ED in AN adolescent subjects could contribute to a more severe clinical picture with a different response to treatments compared to those with low levels of ED.

The present study supports and suggests the identification of specific phenotypes within the AN population, especially in adolescence, and more widely in developmental age, in order to develop dedicated, evidence-based intervention protocols to identify the most appropriate pharmacological and psychotherapeutic treatment approaches.

## Figures and Tables

**Table 1:** Pharmacological treatments and psychotherapy interventions.

	AN+ED (*n* = 21)	AN−ED (*n* = 19)
**Antidepressants**	14 (66.67%)	11 (57.89%)
Fluoxetine	3 (14.29%)	0 (0.00%)
Sertraline	10 (47.62%)	6 (31.58%)
Fluvoxamine	1 (4.76%)	4 (21.05%)
Paroxetine	0 (0.00%)	1 (5.79%)
**Atypical Antipsychotics**	13 (61.90%)	9 (47.37%)
Risperidone	0 (0.00%)	1 (5.79%)
Aripiprazole	9 (42.86%)	6 (31.58%)
Olanzapine	2 (9.52%)	1 (5.79%)
Quetiapine	2 (9.52%)	1 (5.79%)
**Mood Stabilizers**	7 (33.33%)	3 (15.79%)
Lithium Salts	7 (33.33%)	2 (10.53%)
Gabapentin	0 (0.00%)	1 (5.79%)
**No Pharmacotherapy**	2 (9.52%)	4 (21.05%)
**Individual Psychotherapy**	21 (100%)	19 (100%)
**Familiar Psychotherapy**	11 (52.38%)	12 (63.16%)

**Table 2:** Clinical features of the two groups at T0 and at T1.

	T0				Cohen’s d	T1				Cohen’s d
	AN+ED	AN−ED	t	*p*		AN+ED	AN−ED	t	*p*	
**age (years)**	13.95 (1.20)	14.95 (1.65)	2.195	0.034	0.69					
**VCI**	107.37 (11.81)	109.00 (14.51)	0.380	0.706	0.12					
**PRI**	109.74 (11.30)	117.21 (14.81)	1.749	0.089	0.57					
**WMI**	93.16 (12.59)	102.63 (14.08)	2.186	0.035	0.71					
**PSI**	99.58 (12.55)	96.89 (11.78)	−0.680	0.501	0.22					
**FSIQ**	104.63 (12.38)	109.79 (14.83)	1.164	0.252	0.38					
**CGI-S**	5.52 (0.60)	4.95 (0.52)	−3.238	0.003	1.01	4.67 (1.06)	4.33 (1.08)	−0.996	0.340	0.32
**C-GAS**	32.86 (13.65)	42.89 (10.45)	2.625	0.013	0.82	48.33 (11.87)	53.06 (11.52)	1.255	0.217	0.40
**BMI (kg/m^2^)**	15.46 (1.89)	16.81 (2.09)	2.155	0.038	0.68	18.16 (1.63)	17.97 (1.56)	−0.358	0.723	0.12
**BUT-WP**	4.32 (0.52)	3.46 (1.31)	−2.685	0.013	0.88	3.41 (1.54)	2.97 (1.56)	−0.888	0.380	0.28
**BUT-BIC**	4.18 (0.57)	3.28 (1.29)	−2.788	0.010	0.91	3.17 (1.65)	2.88 (1.78)	−0.529	0.600	0.17
**EDI-DT**	26.62 (3.34)	22.26 (7.50)	−2.332	0.028	0.76	19.43 (12.28)	17.11 (11.20)	−0.611	0.545	0.20
**EDI-B**	6.81 (5.65)	2.95 (3.24)	−2.613	0.013	0.82	5.05 (5.25)	3.83 (5.18)	−0.724	0.473	0.23
**EDI-BD**	33.86 (6.26)	26.95 (9.72)	−2.699	0.010	0.85	27.81 (13.82)	23.83 (12.33)	−0.941	0.353	0.30
**EDI-ID**	26.24 (6.56)	12.21 (8.73)	−5.779	<0.001	1.92	20.52 (9.46)	10.83 (9.28)	−3.217	0.003	1.04
**EDI-ED**	16.67 (5.58)	6.37 (5.05)	−6.098	<0.001	1.93	11.95 (7.85)	4.06 (3.90)	−4.063	<0.001	1.25
**CHT**	15.90 (2.81)	8.84 (3.59)	−6.960	<0.001	2.20	14.71 (4.57)	7.78 (4.76)	−4.634	<0.001	1.48
**YSR-Int**	75.14 (9.40)	64.84 (12.27)	−2.998	0.005	0.95	69.67 (13.02)	61.33 (7.41)	−2.399	0.022	0.77
**YSR-Ext**	56.76 (6.59)	49.53 (5.70)	−3.694	0.001	1.16	55.95 (5.89)	47.50 (5.32)	−4.669	<0.001	1.50
**YSR-A/D**	79.95 (9.55)	69.89 (11.06)	−3.393	0.002	0.97	76.86 (14.35)	65.44 (10.44)	−2.797	0.008	0.90
**YSR-W/D**	77.71 (11.14)	69.21 (11.90)	−2.335	0.025	0.74	68.29 (14.34)	61.94 (8.16)	−1.658	0.106	0.53

VCI = Verbal Comprehension Index, PRI = Perceptual Reasoning Index, WMI = Working Memory Index, PSI = Processing Speed Index, FSIQ = Full-Scale Intelligence Quotient, CGI-S = Clinic Global Impression—Severity, C-GAS = Children’s Global Assessment Scale, BMI = Body Mass Index, BUT-WP = Body Uneasiness Test—Weight Phobia, BUT-BIC = Body Uneasiness Test—Body Image Concerns, EDI-DT = Eating Disorder Inventory—Drive for Thinness, EDI-B = Eating Disorder Inventory—Bulimia, EDI-BD = Eating Disorder Inventory—Body Dissatisfaction, EDI-ID = Eating Disorder Inventory—Interoceptive Deficits, EDI-ED = Eating Disorder Inventory—Emotional Dysregulation; CHT = Cyclothymic-Hypersensitive Temperament; YSR-Int = Youth Self Report—Internalizing Problems; YSR-Ext = Youth Self Report—Externalizing Problems; YSR-A/D = Youth Self Report—Anxious/Depressed; YSR-W/D = Youth Self Report—Withdrawn/Depressed.

**Table 3:** Principal component comparison between the two groups.

	F	*p*	Partial Eta-Squared
**BMI**			
Time	4.693	0.037	0.115
Time × Group	3.224	0.081	0.082
Group	0.104	0.748	0.003
Age	10.507	0.003	0.226
**CGI-S**			
Time	4.638	0.038	0.114
Time × Group	0.087	0.770	0.002
Group	0.087	0.770	0.002
Age	2.705	0.109	0.070
**C-GAS**			
Time	7.757	0.008	0.177
Time × Group	0.741	0.395	0.020
Group	5.120	0.030	0.125
Age	0.519	0.476	0.014
**EDI-DT**			
Time	3.270	0.079	0.083
Time × Group	0.011	0.917	0.001
Group	3.538	0.068	0.089
Age	2.624	0.114	0.068
**EDI-B**			
Time	1.960	0.170	0.052
Time × Group	4.288	0.046	0.106
Group	5.432	0.025	0.131
Age	3.597	0.066	0.091
**EDI-BD**			
Time	1.836	0.184	0.049
Time × Group	0.166	0.686	0.005
Group	0.166	0.686	0.005
Age	1.257	0.270	0.034
**EDI-ID**			
Time	0.309	0.582	0.009
Time × Group	1.550	0.221	0.041
Group	21.356	<0.001	0.372
Age	0.026	0.873	0.001
**EDI-ED**			
Time	2.283	0.140	0.060
Time × Group	0.372	0.546	0.010
Group	34.513	<0.001	0.489
Age	0.189	0.667	0.005
**BUT-WP**			
Time	3.020	0.091	0.077
Time × Group	0.324	0.573	0.009
Group	6.194	0.018	0.147
Age	3.892	0.056	0.098
**BUT-BIC**			
Time	3.020	0.091	0.077
Time × Group	0.324	0.573	0.009
Group	6.194	0.018	0.147
Age	3.892	0.056	0.098

BMI = Body Mass Index, CGI-S = Clinic Global Impression—Severity, C-GAS = Children’s Global Assessment Scale, EDI-DT = Eating Disorder Inventory—Drive for Thinness, EDI-B = Eating Disorder Inventory—Bulimia, EDI-BD = Eating Disorder Inventory—Body Dissatisfaction, EDI-ED = Eating Disorder Inventory—Emotional Dysregulation, EDI-ID = Eating Disorder Inventory—Interoceptive Deficits, BUT-WP = Body Uneasiness Test—Weight Phobia, BUT-BIC = Body Uneasiness Test—Body Image Concerns.

## Data Availability

The data presented in this study are available on request from the corresponding author due to privacy.
